# Prevalence and effects of sleep-disordered breathing on middle-aged patients with sedative-free generalized anxiety disorder: A prospective case-control study

**DOI:** 10.3389/fpsyt.2022.1067437

**Published:** 2023-01-09

**Authors:** Tien-Yu Chen, Yen-Ying Kung, Hsiao-Ching Lai, Li-Ang Lee, I-An Jen, Hsin-An Chang, Chia-Yu Liu, Terry B. J. Kuo, Cheryl C. H. Yang

**Affiliations:** ^1^Institute of Brain Science, National Yang Ming Chiao Tung University, Taipei, Taiwan; ^2^Department of Psychiatry, Tri-Service General Hospital, School of Medicine, National Defense Medical Center, Taipei, Taiwan; ^3^Sleep Research Center, National Yang Ming Chiao Tung University, Taipei, Taiwan; ^4^School of Medicine, Institute of Traditional Medicine, National Yang Ming Chiao Tung University, Taipei, Taiwan; ^5^Center for Traditional Medicine, Taipei Veterans General Hospital, Taipei, Taiwan; ^6^Faculty of Medicine, School of Medicine, National Yang Ming Chiao Tung University, Taipei, Taiwan; ^7^Department of Otorhinolaryngology, Head and Neck Surgery, Sleep Center, Linkou Chang Gung Memorial Hospital, Taoyuan, Taiwan; ^8^Department of Public Health, College of Medicine, National Yang Ming Chiao Tung University, Taipei, Taiwan; ^9^Tsoutun Psychiatric Center, Ministry of Health and Welfare, Nantou, Taiwan; ^10^Clinical Research Center, Taoyuan Psychiatric Center, Ministry of Health and Welfare, Taoyuan, Taiwan; ^11^Brain Research Center, National Yang Ming Chiao Tung University, Taipei, Taiwan

**Keywords:** generalized anxiety disorder, sleep-disordered breathing, heart rate variability, home sleep apnea test, autonomic function

## Abstract

**Objective:**

Generalized anxiety disorder (GAD) and sleep-disordered breathing (SDB) share similar symptoms, such as poor sleep quality, irritability, and poor concentration during daily activities. This study aims to investigate the proportion of undiagnosed SDB and its impacts on anxiety severity and autonomic function in newly diagnosed, sedative-free GAD patients.

**Methods:**

This prospective case-control study included newly diagnosed GAD patients and control participants with matched age, sex, and body mass index (BMI) in Taiwan. All participants completed questionnaires for sleep and mood symptoms and a resting 5-min heart rate variability (HRV) examination during enrollment. The participants also used a home sleep apnea test to detect SDB. An oxygen desaturation index (ODI) ≥ 5 was considered indicative of SDB.

**Results:**

In total, 56 controls and 47 newly diagnosed GAD participants (mean age 55.31 ± 12.36 years, mean BMI 23.41 ± 3.42 kg/m^2^) were included. There was no significant difference in the proportion of undiagnosed SDB in the control and sedative-free GAD groups (46.43 vs. 51.06%). Sedative-free GAD patients with SDB scored significantly higher on Beck Anxiety Inventory (23.83 ± 11.54) than those without SDB (16.52 ± 10.61) (*p* < 0.001). Both control and sedative-free GAD groups with SDB had worse global autonomic function than the control group without SDB, as evidenced by the HRV results (*p* < 0.05 for all).

**Conclusion:**

Average age 55 years and mean BMI 23 kg/m^2^ patients with GAD and matched controls had an undiagnosed SDB prevalence of approximately 50%. SDB correlated with worsening anxiety severity and reduced cardiac autonomic function. Moreover, age and BMI were considered major risk factors for predicting undiagnosed SDB.

## Introduction

Generalized anxiety disorder (GAD) is a highly prevalent anxiety disorder characterized by excessive worry associated with restlessness, muscle tension, fatigue, irritability, problems with sleep, and poor concentration (1). GAD is highly comorbid with other psychiatric conditions but also with indicators of pain, stress, and health care utilization. GAD has a significant negative impact on both individuals and society (2).

Sleep-disordered breathing (SDB) is a prevalent sleep disorder characterized by episodic upper airway obstruction during sleep (3). The symptoms of SDB include sleep disturbance, loud snoring, increased sympathetic activity during sleep, daytime sleepiness, poor concentration, irritability, and falling asleep during routine activities (4). Diagnosis of SDB at an early stage may be challenging owing to limit diagnostic tools, and a high prevalence of undiagnosed SDB in Asians and Caucasians has been previously reported (5–8).

Generalized anxiety disorder and sleep-disordered breathing share similar symptoms, such as poor sleep quality, irritability, and poor concentration during daily activities. Some evidence has reported a bidirectional relationship between anxiety disorders and SDB (9, 10). A recent retrospective study in Taiwan reported that those patients with anxiety disorders had an adjusted odds ratio of 1.864 in SDB comorbidity, and that the number of anxiety disorder diagnoses was higher only before, but not after, SDB diagnosis (11). Previous studies often examined SDB in patients with mood disorders, and some participants may have taken antidepressants or sedative medications for a period of time (8, 12). However, since many antidepressants and sedative medications could affect respiratory muscle strength and the presence of SDB, SDB should be assessed in sedative-free anxiety patients to clarify the association between anxiety disorders and SDB (13).

In addition, heart rate variability (HRV) is a physiological phenomenon of variation in the time interval between heartbeats. According to previous studies, patients with GAD may have lower resting-state HRV for parasympathetic activity (14), and patients with SDB may have reduced total HRV in resting HRV measurement (15–17). However, no previous studies have examined the role of HRV in the correlation between GAD and SDB.

In the present study, we aimed to investigate the proportion of sedative-free patients with an initial diagnosis of GAD with undiagnosed SDB, whether comorbid SDB influenced the patients’ GAD symptoms, autonomic function, and whether there were relevant risk factors that would cause the comorbidity of GAD and SDB in Taiwan.

## Methods

### Participants and procedures

This prospective case-control study included newly diagnosed, sedative-free GAD patients and controls with matched age, sex, and body mass index (BMI) in Taiwan. We included patients with newly diagnosed GAD enrolled by a board-certificated psychiatrist from Tri-Service General Hospital (Taipei, Taiwan), and matched controls from the local community in Northern Taiwan. The research protocol was reviewed and approved by the Institutional Review Boards of the Tri-Service General Hospital (TSGH) (IRB No. 2-107-05-046) and Taipei Veterans General Hospital (TVGH) (IRB No. 2020-01-026CCF#1). All patients/participants provided written informed consent before participation, and this study complied with the Declaration of Helsinki.

### Inclusion and exclusion criteria

Generalized anxiety disorder patients were enrolled in the TSGH if they [1] were between 45 and 65 years old; [2] were diagnosed by a board-certificated psychiatrist with Beck Anxiety Inventory (BAI) scores ≥ 8 (18); [3] had not taken sedative central nervous system drugs (including benzodiazepine receptor agonists, antipsychotics, and sedative antidepressants) within 2 weeks before the study; [4] had a habitual bedtime between 8:30 p.m. and midnight; [5] had a BMI between 18 and 34; [6] were willing to sign the informed consent forms.

Patients who [1] changed their sleeping routine 3 months prior to the study due to working conditions, who [2] traveled across more than three time zones within the prior week of the study, who [3] had other significantly unstable physical or mental states that could affect the sleep and wake functions, and who [4] have arrhythmia or used any anti-arrhythmia medications such as beta blockers were excluded from the study.

The control group comprised participants aged between 45 and 65 years old, had BAI scores <8, had a habitual bedtime between 8:30 p.m. and midnight, and had a BMI between 18 and 34 from the community in Northern Taiwan. They were free of major physical illnesses, including major cardiovascular events, arrhythmia, liver or kidney disease, metabolic disorders, malignancy, neurological disorders, and mental disorders. No participants had been taking any medications, as determined by self-reporting, for at least 1 month prior to the study.

### Procedures

From September 2019 to February 2021, we enrolled 113 subjects, 51 patients with newly diagnosed GAD, and 62 age, sex, and BMI matched controls. The participants then underwent a resting 5-min HRV examination using a handheld electrocardiography (ECG) monitor (WG-101, Wegene, New Taipei, Taiwan). The participants were asked to sit quietly for 20 min before the 5-min ECG monitoring, which was performed with a lead I electrocardiogram while the subjects sat quietly and breathed normally (19). The HRV parameters were analyzed serially. First, we identified each QRS complex, and the R-point of each QRS complex was defined as the time point of each heartbeat. Second, the interval between two R points (R-R interval) was estimated as the interval between the current and latter R points. Third, the computation of the spectrum was calculated by fast Fourier transform (FFT), and the resulting spectrum was corrected from the sampling and Hamming window. Finally, the power spectrum was quantified into various frequency domains: very low frequency (VLF) (0.003–0.04 Hz), low frequency (LF) (0.04–0.15 Hz), and high frequency (HF) (0.15–0.4 Hz). LF and HF were normalized by the percentage of total power minus VLF into LF% and HF%. In addition, the LF/HF ratio can be used as a representation of sympathovagal balance. Total power (TP) indicated the global autonomic function. Time parameters, such as the standard deviation of normal-to-normal beats (SDNN), were analyzed simultaneously (20).

The participants were required to complete a set of Chinese-version questionnaires, including Pittsburgh Sleep Quality Index (PSQI) (21), Epworth Sleepiness Scale (ESS) (22), 21-item version of Beck Anxiety Inventory (BAI) (18), and Beck Depression Inventory (BDI) (23), to evaluate their sleep quality, daytime sleepiness, and severity of anxiety and depression, respectively.

When they completed the above surveys, the participants were given instructions on how to use the fingertip pulse oximeter (AT101l, Leadtek Research Inc., New Taipei, Taiwan) so that they could record their physical signals for two consecutive nights. There are four different types of sleep studies, leveling from type I to type IV. Type I sleep study, or polysomnography, is the gold standard for diagnosing SDB with a minimum of seven electronic records of signals. However, it requires participants to stay overnight in a sleep laboratory with technicians in attendance, which is a time-consuming and expensive process. Type II sleep study utilizes portable polysomnography. Type III monitor requires a minimum of four signals, and type IV sleep study requires a minimum of one signal. Type III and type IV sleep studies are frequently used as home sleep apnea tests (HSAT), which are more convenient and cost-effective than in-lab test studies. Due to the limitations of PSG and the fact that the present study was a major community study, we used fingertip pulse oximeters, a type IV HSAT that has an 81.25% accuracy in diagnosing SDB, as an alternative SDB screening system. An ODI ≥ 5 was considered to signify SDB in this study (24). Although all participants used the fingertip pulse oximeter for two nights, only the data from the second night were considered in the study because the participants could be unfamiliar with the tasks at the first night, resulting in false or inaccurate reports (25).

### Statistical analyses

All statistical analyses were performed using SPSS software (v. 28; IBM Corporation, New York, USA), and a *P*-value < 0.05 was considered statistically significant. Group differences were evaluated by Pearson’s chi-square test and one-way analysis of variance (ANOVA) with the Bonferroni *post-hoc* test. Correlation analyses of BAI and ODI with other variables were performed using Pearson’s correlation coefficient (*r*). Linear regression models were built to identify the variables that were most significantly associated with BAI and ODI. Specifically, we used the backward stepwise method for the linear regression analyses; the method starts with a model that contains all the selected variables, removes the least significant variable one at a time, and continues to remove variables until a pre-specified stopping rule is reached or until no variable is left in the model.

## Results

### Demographic of participants

After data collection, 10 participants with missing data were excluded. A total of 103 valid participants, with 56 controls and 47 GAD participants (42 men and 61 women), were included in the final analyses. The mean age of the enrolled participants was 55.31 ± 12.36 years, and the mean BMI was 23.41 ± 3.42 kg/m^2^ (mean ± SD).

Table 1 outlines the participants’ characteristics. The sedative-free GAD participants and control participants were well matched in age, sex, and BMI. We also found that the sedative-free GAD group had higher scores of PSQI, BAI, and BDI. For 5-min resting HRV, sedative-free GAD group had lower TP, LF%, and LF/HF. In addition, we found that there was no difference in ODI between sedative-free GAD and control groups. We used ODI ≥ 5 as a SDB diagnostic criterion to classify the participants into four subgroups to further evaluate the impact of ODI in sedative-free GAD and control participants: Group 1/control and ODI < 5 (*n* = 30, 29.1%), Group 2/control and ODI ≥ 5 (*n* = 26, 25.3%), Group 3/GAD and ODI < 5 (*n* = 23, 22.3%), and Group 4/GAD and ODI ≥ 5 (*n* = 24, 23.3%). There were no statistical differences among the four groups in terms of age, sex, or BMI (Table 2).

**TABLE 1:** Comparison of demographic data and clinical characteristics of the study participants.

	Control	Sedative-free GAD	*P*
Sex			0.266
Male	26 (46.4)	16 (34.0)	
Female	30 (53.6)	31 (66.0)	
Total, N	56 (100.0)	47 (100.0)	
Age, years	54.51 ± 13.53	56.30 ± 10.80	0.466
BMI, kg/m^2^	23.01 ± 3.14	23.90 ± 3.72	0.196
≤25	46 (82.1)	36 (76.6)	0.348
≥25	10 (17.9)	11 (23.4)	
**Scales**			
ODI	8.219.26	8.22 ± 8.86	0.994
PSQI	7.53 ± 3.73	13 ± 3.54	<0.001**
Sleep duration (hours)	5.92 ± 1.50	5.20 ± 1.71	0.039*
ESS	7.42 ± 4.96	6.64 ± 4.82	0.427
BAI	2.93 ± 2.60	20.42 ± 11.54	<0.001**
BDI	4.95 ± 6.48	17.50 ± 11.29	<0.001**
TP	6.67 ± 1.01	6.20 ± 1.05	0.024*
LF	5.16 ± 1.27	4.78 ± 1.27	0.137
HF	4.33 ± 1.40	4.53 ± 1.52	0.495
LF%	63.91 ± 17.68	51.98 ± 20.90	0.002*
LF/HF	0.86 ± 0.85	0.25 ± 1.04	0.001*
SDNN	35.00 ± 18.21	29.08 ± 13.64	0.071

Data are presented as n (%) or mean ± standard deviation. *P <0.05; **P <0.001. GAD, generalized anxiety disorder; BMI, body mass index; ODI, oxygen desaturation index; PSQI, pittsburgh sleep quality index; ESS, epworth sleepiness scale; BAI, beck anxiety inventory; BDI, beck depression inventory; TP, total power; LF, low-frequency power; HF, high-frequency power; LF%, normalized low-frequency power; LF/HF, ratio of LF-to-HF power; SDNN, standard deviation of N-N intervals.

**TABLE 2:** Comparison of demographic data and clinical characteristics of the study participants by ODI subgroups.

	Control		Sedative-free GAD		*P*	
			
Demographics	ODI < 5 (1) (53.57)	ODI ≥ 5 (2) (46.43)	ODI < 5 (3) (48.94)	ODI ≥ 5 (4) (51.06)	0.944	
Sex					0.062	
Male	18 (60.0)	8 (30.7)	9 (39.1)	7 (29.2)		
Female	12 (40.0)	18 (69.3)	14 (60.9)	17 (70.8)		
Total, N	30 (100.0)	26 (100.0)	23 (100.0)	24 (100.0)		
Age, years	51 ± 22.43	59 ± 11	55 ± 23.1	58 ± 6	0.055	
BMI, kg/m^2^	22.43 ± 2.26	23.69 ± 3.86	23.1 ± 2.53	24.62 ± 4.47	0.119	
<25	28 (93.3)	18 (72.0)	19 (82.6)	17 (70.8)	0.124	
≥25	2 (6.7)	7 (28.0)	4 (17.4)	7 (29.2)		

**Scales**	**ODI < 5 (1)**	**ODI ≥ 5 (2)**	**ODI < 5 (3)**	**ODI ≥ 5 (4)**	***P***	**Bonferroni**

ODI	2.48 ± 1.45	14.58 ± 10.11	2.40 ± 1.47	13.57 ± 9.46	<0.001**	2, 4 > 1, 3
PSQI	6.92 ± 4.20	7.96 ± 3.02	12.62 ± 4.23	13.33 ± 2.78	<0.001**	3, 4 > 1, 2
Sleep duration (hours)	6.02 ± 1.64	5.97 ± 1.10	5.38 ± 1.60	4.94 ± 1.86	0.076	
ESS	5.77 ± 4.80	9.26 ± 4.55	5.20 ± 3.74	7.83 ± 5.35	0.010*	2 > 1, 3
BAI	2.60 ± 2.57	3.3 ± 2.64	16.52 ± 10.61	23.83 ± 11.54	<0.001**	4 > 3 > 1, 2
BDI	4.63 ± 5.00	5.3 ± 7.90	15.05 ± 9.21	19.54 ± 12.59	<0.001**	3, 4 > 1, 2
TP	7.02 ± 1.08	6.28 ± 0.77	6.28 ± 1.07	6.13 ± 1.06	0.005*	1 > 2, 4
LF	5.72 ± 1.34	4.53 ± 0.85	4.86 ± 1.43	4.75 ± 1.14	0.002*	1 > 2, 4
HF	4.88 ± 1.37	3.72 ± 1.17	4.57 ± 1.40	4.49 ± 1.65	0.020*	1 > 2
LF%	65.04 ± 15.59	62.66 ± 19.98	51.88 ± 19.06	52.08 ± 22.87	0.024*	
LF/HF	0.91 ± 0.67	0.81 ± 1.03	0.25 ± 0.88	0.25 ± 1.19	0.017*	
SDNN	41.00 ± 22.10	28.33 ± 9.01	30.62 ± 15.07	27.68 ± 12.33	0.006*	1 > 2, 4

Data are presented as n (%) or mean ± standard deviation. *P <0.05; **P <0.001. GAD, generalized anxiety disorder; ODI, oxygen desaturation index; BMI, body mass index; PSQI, pittsburgh sleep quality index; ESS, epworth sleepiness scale; BAI, beck anxiety inventory; BDI, beck depression inventory; TP, total power; LF, low-frequency power; HF, high-frequency power; LF%, normalized low-frequency power; LF/HF, ratio of LF-to-HF power; SDNN, standard deviation of N-N intervals.

For self-reported questionnaires as PSQI, ESS, BAI, and BDI, the statistical differences were found among the four groups in all four surveys (Table 2). For the PSQI and BDI outcomes, Groups 3 and 4 were found to have significantly higher scores than Groups 1 and 2 (*p* < 0.001 for both). Regarding ESS, Group 2 scored higher than Groups 1 and 3 (*p* = 0.010). In the case of BAI, Group 4 scored higher than Group 3, which together scored higher than the two control groups (*p* < 0.001). In general, the GAD participants were more anxious than the control participants, which was consistent with their diagnosis, while participants with GAD and ODI ≥ 5 had the highest level of anxiety. From the ODI analysis (*p* < 0.001), Groups 2 and 4 scored higher than the other two groups, corresponding to how the four small groups were formed.

Among the six HRV indices in the four groups, participants in Group 1 were found to have (1) significantly higher TP (*p* = 0.005), LF (*p* = 0.002), and SDNN (*p* = 0.006) than those in Groups 2 and 4, and (2) a significantly higher HF than participants in Group 2 (*p* = 0.020). Although significant differences were found among the groups for LF% (*p* = 0.024) and LF/HF (*p* = 0.017), we failed to detect the exact differences with the Bonferroni *post-hoc* test.

### Pittsburgh sleep quality index components

The items of PSQI were grouped into seven component scores: subjective sleep quality, sleep latency, sleep duration, habitual sleep efficiency, sleep disturbances, use of sleep medication, and daytime dysfunction. Table 3 shows the results of the PSQI in the different groups. Groups 3 and 4 (the GAD group) scored significantly higher than groups 1 and 2 (the control group) in terms of sleep quality, sleep latency, and use of sleeping medication (*p* < 0.001 for all). There were no significant differences found across groups in the components “sleep duration” and “habitual sleep efficiency.” In general, participants in the sedative-free GAD group had worse sleep quality and conditions.

**TABLE 3:** Comparison of PSQI result by component and group.

	Control		Sedative-free GAD			
Components	ODI < 5 (1)	ODI ≥ 5 (2)	ODI < 5 (3)	ODI ≥ 5 (4)	*P*	Bonferroni
Sleep quality	1.26 ± 0.98	1.17 ± 0.65	2.25 ± 0.72	2.35 ± 0.78	<0.001**	3, 4 > 1, 2
Sleep latency	1.15 ± 0.86	1.22 ± 0.80	2.15 ± 0.88	1.87 ± 0.97	<0.001**	3, 4 > 1, 2
Sleep duration	1.59 ± 1.08	1.91 ± 0.90	1.90 ± 1.07	2.17 ± 0.83	0.227	
Habitual sleep efficiency	1.00 ± 1.29	1.26 ± 1.18	1.70 ± 1.30	1.78 ± 1.13	0.102	
Sleep disturbance	0.96 ± 0.44	1.35 ± 0.57	1.55 ± 0.69	1.70 ± 0.70	<0.001**	3, 4 > 1
Use of sleeping medication	0.48 ± 0.94	0.30 ± 0.88	2.35 ± 1.09	2.17 ± 1.34	<0.001**	3, 4 > 1, 2
Daytime dysfunction	0.52 ± 0.58	0.74 ± 0.69	1.00 ± 0.80	1.26 ± 0.92	0.005*	4 > 1

Data are presented as mean ± standard deviation. *P < 0.05; **P < 0.001. PSQI, pittsburgh sleep quality index; GAD, generalized anxiety disorder; ODI, oxygen desaturation index.

### Correlational analysis between Beck anxiety inventory and other variables in sedative-free generalized anxiety disorder participants

As shown in Supplementary Table 1, we included 14 variables (sex, age, BMI, PSQI, sleep duration, ESS, BAI, BDI, LF, HF, TP, LF%, LF/HF, and SDNN) to observe their correlations with BAI. BMI (*r* = 0.301, *p* < 0.05), PSQI (*r* = 0.407, *p* < 0.001), ESS (*r* = 0.609, *p* < 0.001), and BDI (*r* = 0.629, *p* < 0.001) were significantly positively correlated with BAI, whereas age (*r* = −0.317, *p* < 0.05) was significantly negatively correlated with BAI. Sex, sleep duration, ODI, LF, HF, TP, LF%, LF/HF, and SDNN were not associated with BAI in the sedative-free GAD participants.

### Correlational analysis between oxygen desaturation index and other variables in all participants

Due to no difference of ODI between sedative-free GAD and control, we analyzed the correlation between ODI and other variables in all participants. In Supplementary Table 2, we included the same 14 variables as above to observe their correlations with ODI. Age (*r* = 0.215, *p* < 0.05), BMI (*r* = 0.319, *p* < 0.001), and ESS (*r* = 0.226, *p* < 0.05) were positively correlated with ODI, whereas LF (*r* = −0.232, *p* < 0.05), TP (*r* = −0.225, *p* < 0.05), and SDNN (*r* = −0.213, *p* < 0.05) were negatively correlated with ODI. Sex, PSQI, sleep duration, BAI, BDI, HF, LF%, and LF/HF were not related to ODI in all participants.

### Backward stepwise regression model of Beck anxiety inventory in sedative-free generalized anxiety disorder participants

Using the variables that were found to be correlated with BAI, we conducted a backward stepwise regression analysis with BAI as the dependent variable (Table 4). From the correlational analysis, we found that age, BMI, PSQI, ESS, and BDI were significantly correlated with BAI. Consequently, these five variables were included in the initial model. In addition, we added sex and ODI as independent variables in the first model as we considered them to be crucial factors. After three rounds of backward selection, in the final model, age (*p* = 0.009), PSQI (*p* = 0.018), ESS (*p* = 0.004), and BDI (*p* = 0.034) were concluded as the most influential predictors of BAI in the sedative-free GAD participants.

**TABLE 4:** Backward stepwise regression analysis of BAI with related variables in sedative-free GAD participants.

Model	Variable	B	SE	*P*
1	Sex	–5.555	2.629	0.042*
	Age	–0.370	0.127	0.006*
	BMI	0.580	0.340	0.097
	PSQI	1.017	0.367	0.009*
	ESS	1.034	0.307	0.002*
	BDI	0.196	0.150	0.199
	ODI	–0.149	0.137	0.284
2	Sex	–5.050	2.594	0.059
	Age	–0.362	0.127	0.007*
	BMI	0.471	0.326	0.157
	PSQI	0.992	0.368	0.010*
	ESS	0.945	0.296	0.003*
	BDI	0.229	0.147	0.128
3	Sex	–4.604	2.612	0.086
	Age	–0.355	0.129	0.009*
	PSQI	0.910	0.368	0.018*
	ESS	0.921	0.300	0.004*
	BDI	0.305	0.139	0.034*

*P < 0.05. BMI, body mass index; PSQI, pittsburgh sleep quality index; ESS, epworth sleepiness scale; BDI, beck depression inventory; ODI, oxygen desaturation index.

### Backward stepwise regression model of oxygen desaturation index in all participants

Based on the variables that were found to be correlated with ODI (BMI, ESS, LF, TP, and SDNN), we conducted a backward stepwise regression analysis with ODI as the dependent variable (Table 5). We also added sex as an independent variable in the first model as we considered it a crucial factor (11). After five rounds of backward selection, in the final/6th model, age (*p* = 0.016) and BMI (*p* = 0.000) were found to be the most influential predictors of ODI.

**TABLE 5:** Backward stepwise regression analysis of ODI with related variables in all participants.

Model	Variable	B	SE	*P*
1	Sex	–2.238	1.982	0.262
	Age	0.083	0.088	0.346
	BMI	0.860	0.265	0.002*
	ESS	0.264	0.181	0.147
	LF	–0.577	1.608	0.721
	TP	0.189	2.466	0.939
	SDNN	–0.027	0.125	0.833
2	Sex	–2.234	1.971	0.260
	Age	0.085	0.086	0.326
	BMI	0.858	0.261	0.001*
	ESS	0.263	0.179	0.146
	LF	–0.498	1.234	0.687
	SDNN	–0.020	0.095	0.831
3	Sex	–2.293	1.942	0.241
	Age	0.086	0.085	0.313
	BMI	0.854	0.259	0.001*
	ESS	0.266	0.178	0.138
	LF	–0.706	0.756	0.353
4	Sex	–2.400	1.937	0.218
	Age	0.119	0.077	0.127
	BMI	0.899	0.255	0.001*
	ESS	0.256	0.178	0.152
5	Age	0.159	0.071	0.026*
	BMI	0.835	0.250	0.001*
	ESS	0.284	0.177	0.112
6	Age	0.173	0.071	0.016*
	BMI	0.911	0.247	0.000**

*P < 0.05; **P < 0.001. ODI, oxygen desaturation index; BMI, body mass index; ESS, epworth sleepiness scale; LF, low-frequency power; TP, total power; SDNN, standard deviation of N-N intervals.

## Discussion

### Main findings

This is the first case-control study to investigate the association between undiagnosed SDB and sedative-free GAD. Among the participants with an average age of 55 years and mean BMI of 23 kg/m^2^, nearly 50% of individuals in both the sedative-free GAD and control groups had undiagnosed SDB with reduced global autonomic function. Among patients in the sedative-free GAD group, those with undiagnosed SDB had more severe anxiety symptoms than those without SDB. Moreover, the risk factors that best predicted SDB were found to be age and BMI.

### Clinical implications

This study has several noteworthy findings. First, we found undiagnosed SDB in approximately 50% of both sedative-free GAD patients and control participants with a mean age of 55 years and BMI of 23 kg/m^2^. Most previous studies have investigated comorbid depressive and anxious symptoms in patients with SDB (9, 11, 26), but limited data have evaluated SDB in patients with mental disorders. The value of our study is that we not only discovered the comorbidity rates of SDB in newly diagnosed GAD but also found no difference in the SDB comorbidity rates in the control group. Therefore, our study suggests that GAD itself may not cause SDB, and that a high prevalence of SDB was found in middle-aged, and normal weight adults in Taiwan. Further calling attention to the clinical practitioners of the occurrence of SDB in middle-aged and older adults is important. Furthermore, we enrolled sedative-free GAD participants. Therefore, we can avoid the influence of sedative medications for SDB (13). Our study can also bring some insights for clinicians that it may be worth evaluating craniofacial and oropharyngeal characteristics for SDB (27, 28) since normal weight participants had high ratio of SDB in the middle-aged group in Taiwan.

Second, from the PSQI results, it was found that regardless of whether the sedative-free GAD patients had SDB or not, they generally scored higher in the PSQI than the control group, especially for components such as sleep quality, sleep latency, sleep disturbance, and use of sleeping medication, which further implied poor sleep quality. However, based on the ESS results, only the control group with SDB (group 2) scored higher than the other two groups without SDB (group 1 and group 3), where the former had more severe daytime sleepiness problems than the latter, while no significant differences were found between the sedative-free GAD/SDB group and the other groups. A possible reason is that sedative-free GAD patients are prone to hypervigilance during the day, which reduces the symptoms of daytime sleepiness caused by SDB. Therefore, although daytime sleepiness can be a filter for the diagnosis of SDB (4), it may be relatively insignificant for patients with sedative-free GAD.

Third, in terms of the evaluation of anxiety, it can be inferred from the BAI results that sedative-free GAD patients with SDB had the severest level of anxiety across the groups, with the shortest self-reported sleep duration. Accordingly, it may be more difficult to treat anxiety symptoms in patients with GAD and undiagnosed SDB. In addition, poor sleep quality can affect the level of anxiety in SDB patients. Heightened anxiety levels may be causal to the clinical manifestations of undiagnosed SDB, functionally compensatory processes, or purely epiphenomena. However, case-control studies cannot exactly establish a causal relationship between undiagnosed SDB and heightened anxiety. To address these concerns, there is a growing consensus that symptom-pathophysiology relationships may be best tested using a within-subjects design (e.g., in which undiagnosed SDB is experimentally manipulated to determine its impact on anxiety symptoms) (29).

Fourth, we found an impact of undiagnosed SDB on resting HRV measurements. HRV is used to evaluate the cardiac autonomic activity and is widely used as a biomarker in psychiatric studies. The results of HRV presentation for GAD have been controversial in previous studies. One study from Japan reported significantly higher HF in GAD (30), while another from Taiwan showed significantly lower HF in GAD (31). For patients with SDB, a previous study showed that sympathetic function was elevated during sleep and total HRV was reduced in resting HRV measurement (15–17). In Table 1, we found that sedative-free GAD group had a lower global autonomic function (TP) and lower LF% and LF/HF than the control group. When we considered undiagnosed SDB in the control and sedative-free GAD, we found that both the control and sedative-free GAD groups with undiagnosed SDB had lower global autonomic function (TP and SDNN) than the control group without SDB. We also found that sedative-free GAD and GAD/SDB groups had lower LF% and LF/HF than two control groups, but we failed to detect the exact differences from the *post-hoc* test. In addition, we did not find a difference in HF between the sedative-free GAD and control groups, but reduced HF was found in the control with SDB group. The findings of the present study showed that SDB had a significant effect on HRV. Future research on HRV should consider undiagnosed SDB.

Finally, we found that age and BMI were risk factors for undiagnosed SDB in all participants based on the backward stepwise regression model of ODI. The prevalence of SDB increases with age and BMI as reported in previous studies (4, 32). A high prevalence of undiagnosed SDB has been previously reported (5, 6, 33). If patients with SDB have no bed partners, they may be unaware of their sleep conditions. Many researchers showed that both daytime sleepiness and SDB are often diagnosed late or undiagnosed due to a lack of sleep information and limited resources for SDB diagnosis (34–36). Therefore, the present study also reveals the importance of the application of the HSAT in psychiatry, especially in patients with emotional disorders combined with sleep symptoms.

## Limitations

This study has several limitations. First, the diagnosis of SDB was obtained using a type IV HSAT device instead of an in-lab polysomnography examination. However, in-lab polysomnography is costly and time-consuming for prospective studies, and the device we used was reported to have an 81.25% accuracy in SDB diagnosis (37). From the screening perspective, there is a need for in-home screening devices. In the present study, all included participants used the fingertip pulse oximeter for two nights, and the report from the second night was used to avoid first-night effects from influencing the results. Second, we used self-report questionnaires (PSQI, ESS, BAI, and BDI) to investigate participants’ sleep, daytime sleepiness, and mood conditions. The results of these questionnaires may have recall bias, but these instruments have been widely used in the past and our researchers used every effort to ensure the quality of data. Third, the participants enrolled in this study were all mid-age Taiwanese, and the sample size was relatively small, which may have led to the overfitting of the model. Thus, replication studies using a larger, diverse race and age, with an adequately controlled method, are required to validate the current research findings.

## Conclusion

According to the present study, nearly 50% of individuals with an average age of 55 years and mean BMI of 23 kg/m^2^ in both the sedative-free GAD and control groups had undiagnosed SDB with reduced global autonomic function. Among patients with sedative-free GAD, those with undiagnosed SDB had more severe anxiety symptoms than those without. Moreover, age and BMI were considered major risk factors for predicting undiagnosed SDB.

## Data availability statement

The raw data supporting the conclusions of this article will be made available by the authors, without undue reservation.

## Ethics statement

The studies involving human participants were reviewed and approved by the Tri-Service General Hospital (TSGH) (IRB No. 2-107-05-046) and Taipei Veterans General Hospital (TVGH) (IRB No. 2020-01-026CCF). The patients/participants provided their written informed consent to participate in this study.

## Author contributions

T-YC took responsibility for the writing of the manuscript. Y-YK, H-CL, L-AL, I-AJ, H-AC, and C-YL contributed to the study design and concept formation. TK and CY took responsibility for the manuscript collection and submission. All authors contributed to the article and approved the submitted version.
