# Supplementary material for: Prevalence and effects of sleep-disordered breathing on middle-aged patients with sedative-free generalized anxiety disorder: A prospective case-control study
Source: Front Psychiatry. 2023 Jan 9;13:1067437. doi: 10.3389/fpsyt.2022.1067437 (PMC9869375; doi:10.3389/fpsyt.2022.1067437)
Supplement: Supplementary file 1 [file Data_Sheet_1.docx]

**Supplementary Table 1.** Pearson’s correlation coefficient (*r*) of BAI with other variables in GAD participants.

| **BAI** | **Sex** | **Age** | **BMI** | **PSQI** | **Sleep Duration** | **ESS** | **ODI** |
| --- | --- | --- | --- | --- | --- | --- | --- |
|  | $-$0.027 | $-$0.317* | 0.301* | 0.407** | $-$0.195 | 0.609** | 0.059 |
|  | **BDI** | **LF** | **HF** | **TP** | **LF%** | **LF/HF** | **SDNN** |
|  | 0.629** | 0.246 | 0.200 | 0.203 | 0.045 | 0.004 | 0.159 |

Notes: **P*$<$0.05; ***P*$<$0.001.

Abbreviations: BAI, Beck Anxiety Inventory; ODI, Oxygen Desaturation Index; BMI, body mass index; PSQI, Pittsburgh Sleep Quality Index; ESS, Epworth Sleepiness Scale; BDI, Beck Depression Inventory; LF, low-frequency power; HF, high-frequency power; TP, total power; LF%, normalized low-frequency power; LF/HF, Ratio of LF-to-HF power; SDNN, Standard deviation of N-N intervals.

| **Supplementary Table 2** Pearson’s correlation coefficient (*r*) of ODI with other variables in all participants | | | | | | | |
| --- | --- | --- | --- | --- | --- | --- | --- |
| **ODI** | **Sex** | **Age** | **BMI** | **PSQI** | **Sleep Duration** | **ESS** | **BAI** |
|  | $-$0.145 | 0.215* | 0.319** | $-$0.007 | 0.079 | 0.226* | 0.029 |
|  | **BDI** | **LF** | **HF** | **TP** | **LF%** | **LF/HF** | **SDNN** |
|  | $-$0.015 | $-$0.232* | $-$0.163 | $-$0.225* | $-$0.061 | $-$0.075 | $-$0.213* |
| Notes: **P*$<$0.05; ***P*$<$0.001.  Abbreviations: BAI, Beck Anxiety Inventory; ODI, Oxygen Desaturation Index; BMI, body mass index; PSQI, Pittsburgh Sleep Quality Index; ESS, Epworth Sleepiness Scale; BDI, Beck Depression Inventory; LF, low-frequency power; HF, high-frequency power; TP, total power; LF%, normalized low-frequency power; LF/HF, Ratio of LF-to-HF power; SDNN, Standard deviation of N-N intervals. | | | | | | | |
